# Polymorphisms in the *XPC* gene affect urinary bladder cancer risk: a case-control study, meta-analyses and trial sequential analyses

**DOI:** 10.1038/srep27018

**Published:** 2016-06-01

**Authors:** Monica Sankhwar, Satya Narayan Sankhwar, Sandeep Kumar Bansal, Gopal Gupta, Singh Rajender

**Affiliations:** 1 Department of Urology, King George Medical University, Lucknow, India; 2 CSIR-Central Drug Research Institute, Lucknow, India

## Abstract

Compromised activity of the DNA repair enzymes may raise the risk of a number of cancers. We analyzed polymorphisms in the Xeroderma Pigmentosum, Complementation Group C (*XPC*) gene for their correlation with urinary bladder cancer. Ala499Val and Lys939Gln polymorphisms were genotyped in 234 urinary bladder cancer cases and 258 control samples. A significant association between Ala499Val polymorphism and bladder cancer was observed (OR = 1.78, CI = 1.19–2.66, p = 0.005); however, Lys939Gln was unrelated (OR = 0.97, CI = 0.65–1.45, P = 0.89). Further analysis revealed that Ala499Val was a significant risk factor only in the presence of smoking (OR = 2.23, CI = 1.28–3.87, p < 0.004) or tobacco chewing (OR = 2.40, CI = 1.43–4.04, p = 0.0008). To further appraise the association, we undertook meta-analyses on seven studies (2893 cases and 3056 controls) on Ala499Val polymorphism and eleven studies (5064 cases and 5208 controls) on Lys939Gln polymorphism. Meta-analyses corroborated the above results, showing strong association of Ala499Val (OR = 1.54, CI = 1.21–1.97, p = 0.001) but not that of Lys939Gln (OR = 1.13, CI = 0.95–1.34, p = 0.171) with urinary bladder cancer risk. In conclusion, *XPC* Ala499Val substitution increases urinary bladder cancer risk, but Lys939Gln appears to be neutral.

Urinary bladder cancer (UBC) is the fifth most common malignancy in the economically advanced countries^1^. Tobacco smoking is the most important and well-established risk factor for bladder cancer, contributing up to 50% of UBC occurrences in men and 20% in women^2^. Other important risk factors include occupational exposures to aromatic amines, polycyclic aromatic hydrocarbons and polluted drinking water containing arsenic and chlorination byproducts^3^. A common property of these exposures is the presence of carcinogens that can induce DNA damage in the bladder epithelium^2^. The damage caused by cigarette smoke and particulate matter is mainly removed by the nucleotide-excision repair pathway (NER), and to a lesser extent, by the base-excision repair pathway (BER). The NER pathway mainly removes the bulky DNA adducts typically generated from the exposure to polycyclic aromatic hydrocarbons in tobacco smoke^4^. Variations in the DNA repair capacity are genetically determined and DNA polymorphisms, which may result in subtle structural alterations in the DNA repair enzymes, are postulated to modulate cancer risk^5^.

Xeroderma Pigmentosum, Complementation Group C (*XPC*) gene encodes a 940 amino acid protein that plays an important role in the initiation of DNA repair^6^. XPC is an important player of the NER pathway. Experimental studies have shown that XPC binds to the radiation repair 23B (RAD23B) protein to form the XPC-RAD23B complex, which is involved in the DNA damage recognition and the initiation of repair via the NER pathway^7^. Polymorphisms in the *XPC* gene may alter the NER capacity and affect genetic predisposition to cancer. Many studies have shown that polymorphisms in the *XPC* gene are associated with the risk of esophageal squamous cell carcinoma, gastric cardiac adenocarcinoma, squamous cell carcinoma of the head and neck, breast cancer, renal cell carcinoma, urinary bladder cancer, advanced colorectal adenoma, oral squamous cell carcinoma, lung cancer, and pancreatic adenocarcinoma^8^,^9^.

Ala499Val and Lys939Gln polymorphisms in the *XPC* gene have been extensively studied in UBC. Previous studies have reported inconsistent results about the association between these polymorphisms and UBC^4^,^5^,^6^,^8^,^10^,^11^,^12^,^13^,^14^,^15^,^16^,^17^,^18^,^19^,^20^. The inconsistencies between these studies indicate that the association of *XPC* polymorphisms with UBC risk may depend on the type of population as well as the environmental factors and host characteristics. In a case-control study, we have evaluated the correlation of Ala499Val and Lys939Gln polymorphisms with UBC risk and the impact of environmental factors on it. We also performed meta-analyses on all eligible case-control studies to quantitatively evaluate the significance of association between these polymorphism and UBC. The results of meta-analysis were validated by undertaking trial sequential analyses.

## Results

General characteristics of the patients and the pathological details are presented in Table 1. A non-smoker was defined as the one who had never smoked or had smoked fewer than 100 cigarettes in his/her lifetime. Current smokers were currently smoking or had stopped smoking less than one year before being diagnosed with UBC^17^. In total, 234 bladder cancer patients were recruited. The diagnosis was made on the basis of urine cytology, radiological investigation (ultrasound-KUB and CT-scan), and histopathology. The majority of patients presented with transitional cell carcinoma (TCC), followed by squamous cell carcinoma (SCC) and adenocarcinoma, with frequencies of 74%, 14%, and 4%, respectively. Most of the patients were confined to stage T1 (44%), followed by T0 (29%) and T2 (27%). The frequency of grade 1 patients (57%) was followed by grade 2 (43%) and grade 3 (10%). The majority of patients were suffering from painless hematuria (90%) and passage of clots (50%). Other symptoms were dysuria (90%), increased frequency of micturition (90%), and bone pain (12%) in patients having metastasis.

### Case-Control study

#### Ala499Val substitution increases UBC risk

The distribution of genotypes among cases and controls is detailed in Table 2. Genotype data for controls in both the polymorphisms was in the Hardy Weinberg equilibrium. A Major difference was seen in the distribution of alternate genotypes at the Ala499Val locus between cases and controls (Tables 2 and 3). A statistically significant association was observed between Ala499Val and UBC in the dominant model (CT + TT: OR = 1.78, CI = 1.19–2.66, p = 0.005). Analysis using co-dominant models also showed that Ala499Val substitution increased UBC risk [CT; OR = 1.69, CI = 1.10–2.60, p = 0.017, TT; OR = 1.96, CI = 1.20–3.19, p = 0.007].

After stratifying cases as per the habits of smoking and tobacco chewing, we found that the substitution increased UBC risk only in smokers [CT + TT: 2.23(1.28–3.87), p = 0.004] and tobacco-chewers [CT + TT (OR = 2.40, CI = 1.43–4.04, p = 0.0008)]. The association was also seen in the co-dominant models, further supporting that substitution at this locus increased the risk of UBC and that the inheritance followed a dominant model. In the case of non-smokers [1.08 (0.54–2.17), p = 0.84] and tobacco non-chewers [1.04 (0.52–2.08), p = 0.92], no association with the UBC risk was seen. The above results suggested that Ala499Val substitution was a UBC risk factor only in the presence of smoking or tobacco-chewing habits.

#### Lys939Gln substitution does not correlate with UBC risk

No major difference was seen in the distribution of alternate genotypes at this locus between cases and controls (Table 3). Whereas, a minor difference was observed in the frequency of homozygous mutant genotype between cases and controls, it was statistically non-significant (OR = 0.97, CI = 0.65–1.45, P = 0.89). Comparisons using dominant, co-dominant, and recessive models did not show any statistically significant difference between cases and controls (Table 3). Stratification on the basis of the habit of smoking and tobacco chewing also showed no association of Lys939Gln with the risk of UBC.

#### Linkage disequilibrium and haplotype analysis

Ala499Val and Lys939Gln polymorphisms were not in significant linkage disequilibrium with each other (D’ = 0.035, LOD = 0.14, r2 = 0.001). Four haplotypes with a frequency of more than 1% were detected (CC-27.9%, AC-25.8%, AT-23.8%, CT-22.5%). The distribution of all the haplotypes, except AC, was significantly different between case and controls (Table 4). AT and CT haplotypes were more common in the cases (27.8% and 25.9%, respectively) as compared to the controls (20.2% and 19.5%, respectively), suggesting them to be the risk factors for UBC while CC haplotype was more frequent in controls (32.2%) as compared to the cases (23.1%), suggesting it to be protective.

#### Meta-analysis

##### Literature review

**Figure 1:**
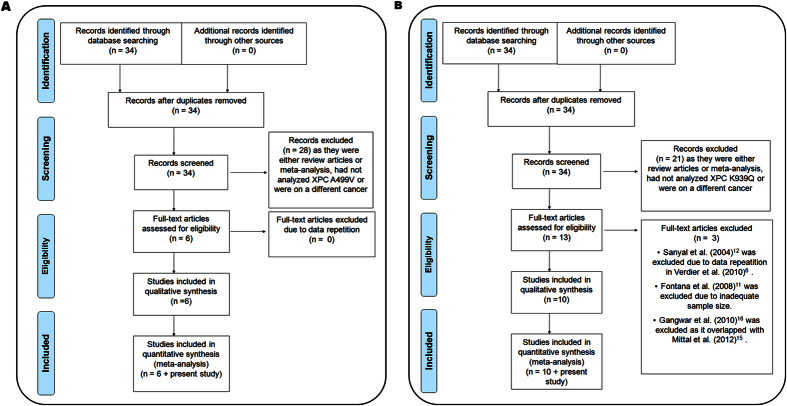
PRISMA flow diagram for inclusion and exclusion of studies in the meta-analysis on Ala499Val (**a**) and Lys939Gln (**b**).

The literature search retrieved a total of 34 relevant articles, which were subjected to systematic screening for inclusion in meta-analysis (Fig. 1). We included a total of seven studies (2893 cases and 3056 controls) for Ala499Val and eleven studies (5064 cases and 5208 controls) for Lys939Gln. In the case of Ala499Val, no full text article was excluded. In the case of Lys939Gln, three full text articles were excluded. Sanyal *et al*.^12^ was excluded as the same samples had been analyzed in Verdier *et al*.^6^. Fontana *et al*.^11^ was excluded as it had used less than 50 samples in either case or control group. Gangwar *et al*.^16^ was excluded as the same samples had been included in Mittal *et al*.^15^. Thus, overall 7957 cases and 8264 controls were included in the meta-analysis (Table 5).

#### Ala499Val increases the risk of UBC

**Figure 2:**
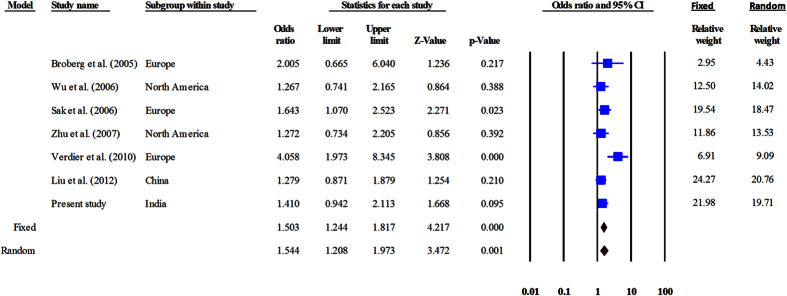
Forest plot for meta-analysis on Ala499Val polymorphism. The Z value shows the degree and direction of relationship, whereas the P value shows the significance of the relationship. The horizontal bar shows the range of OR with a square in the centre, the size of which is directly proportional to the weight given to each study. The direction of projection of the horizontal bar shows the direction of association.

**Figure 3:**
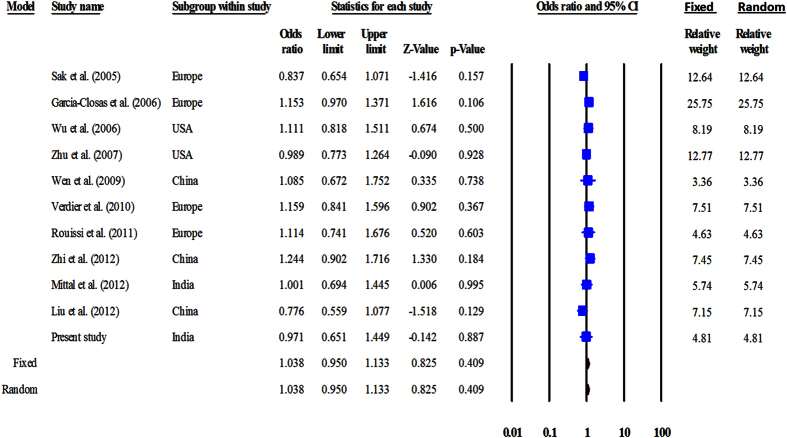
Forest plot for meta-analysis on Lys939Gln polymorphism. The Z value shows the degree and direction of relationship, whereas the P value shows the significance of the relationship. The horizontal bar shows the range of OR with a square in the centre, the size of which is directly proportional to the weight given to each study. The direction of projection of the horizontal bar shows the direction of association.

The genotype data were heterogeneous across all genetic models; hence, the random-effects model was used to perform pooled analysis. Meta-analysis suggested that Ala499Val correlated with UBC risk in the recessive (OR = 1.54, CI = 1.21–1.97, P = 0.001) and co-dominant models (CC vs TT: OR = 1.71, CI = 1.26–2.31, P = 0.001 and CT vs TT: OR = 1.41, CI = 1.16–1.72, P = 0.001) (Fig. 2, Table 6). Lys939Gln did not show correlation with UBC risk in any of the models employed (Fig. 3, Table 6). None of the studies was found to be sensitive enough to significantly affect the outcome of meta-analysis.

#### Europeans carrying XPC variants Ala499Val are at a higher risk of UBC

Looking at the striking differences in the association status among ethnically different populations, we undertook a category-wise analysis on four ethnic groups; Chinese, Indians, Americans, and Europeans using dominant, homozygous, recessive and allelic contrasts. Stratified analysis showed a significant association of Ala499Val substitution with UBC risk only in Europeans across dominant (OR = 1.37, CI = 1.07–1.76, P = 0.011), recessive (OR = 2.21, CI = 1.39–3.50, P = 0.001) and the co-dominant models [CC vs TT: OR = 2.46, CI = 1.41–4.29, P = 0.001 and CT vs TT: OR = 1.90, CI = 1.32–2.75, P = 0.001] (Table 6); however, it was not a significant risk factor in Americans. Subgroup analysis for Lys939Gln revealed that the substitution was not a risk factor in any of the ethnicities (Table 6).

### Trial sequential analysis

**Figure 4:**
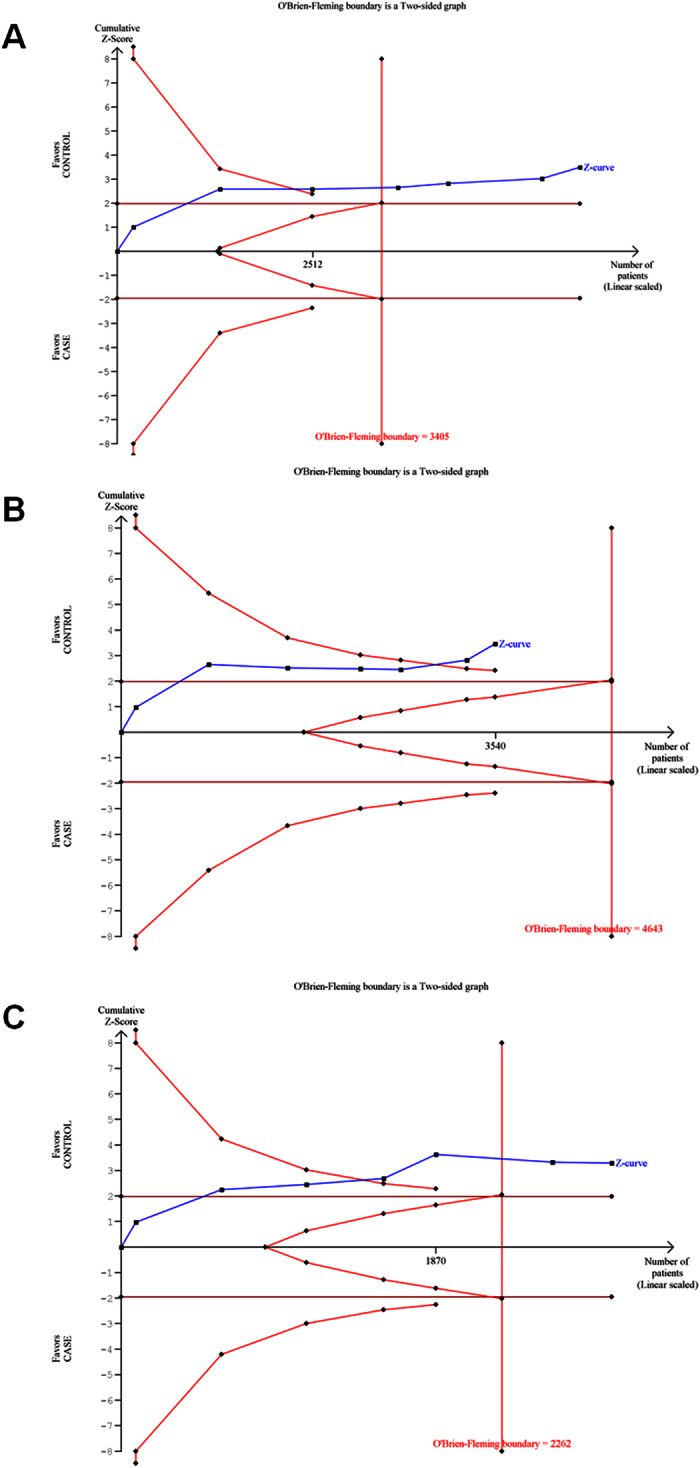
Trial sequential analysis of all studies on Ala499Val polymorphism based on models: (**A**) Recessive, (**B**) Co-dominant CC vs TT, (**C**) Co-dominant CT vs TT.

The results of TSA were consistent with those of the conventional meta-analysis. TSA using co-dominant (CC vs TT and CT vs TT) and recessive models showed that the blue line of cumulative Z-score crossed the red line sloping inwards (significance line of TSA), suggesting a significant association of Ala499Val substitution with the UBC risk (Fig. 4). Further, TSA revealed that meta-analysis had enough number of studies (required sample size = 3405) in the pool to achieve 80% study power as it crossed the O’Brien-Fleming boundary.

In the case of Lys939Gln polymorphism, the results of TSA were similar to those of the conventional meta-analysis, suggesting that Lys939Gln polymorphism is not significantly associated with the UBC risk. In none of the models (dominant, recessive and co-dominant), the blue line of cumulative Z-score crossed the significance line of TSA. Further, TSA revealed that meta-analysis had enough number of studies (required sample size = 2974) in the pool to achieve 80% study power as it crossed the O’Brien-Fleming boundary.

## Discussion

In this study, we analyzed two polymorphisms in the *XPC* gene for their correlation with the UBC risk. Single locus analysis revealed a strong association of Ala499Val substitution with UBC risk, supporting the previous findings on the various ethnic populations^6^,^10^. Nevertheless, a few studies have reported that Ala499Val substitution is not related to UBC risk^13^,^17^,^18^,^20^. We pooled all eligible studies to perform meta-analyses, which suggested a significant association of Ala499Val substitution with increased UBC risk. The association was confirmed by the TSA, further strengthening the conclusion that Ala499Val substitution correlates with an increased risk of UBC. In sub-group analysis, we found that Ala499Val is a significant risk factor in Europeans, but not in Americans. A499V has appeared to be a strong risk factor for cancer as evidenced by a number of recent studies on various cancers^7^. Our finding that Lys939Gln does not affect UBC risk is supported by a number of previous studies 5,8,11,13,14,15,18,19. On the other hand, only a few previous studies reported Lys939Gln to be a risk factor^4^,^6^,^21^, which may be due to ethnic differences. The TSA analysis confirmed that Lys939Gln polymorphism is unrelated to UBC risk, as suggested by the conventional meta-analysis. Subgroup analysis on the basis of ethnicity revealed that Lys939Gln substitution is not a risk factor in any of the ethnic populations.

It is known that smoking and tobacco chewing increase the risk of cancer in general. A few previous studies suggested that the smokers harboring mutated *XPC* genotypes were at an increased risk of UBC when compared to non-smokers^4^,^18^,^19^, but no association was observed between Lys939Gln and increased bladder cancer risk in smokers in a few other studies^2^,^14^,^15^. We found that Ala499Val was a risk factor only in the presence of smoking or tobacco-chewing. Our study strengthens the hypothesis that genetic variations may significantly increase cancer risk in combination with the environmental factors. However, we found Lys939Gln to be a neutral polymorphism irrespective of smoking or tobacco chewing status. Previous studies have demonstrated that XPC deficiency is an important contributing factor in bladder tumor progression and bladder cancer cell drug resistance^22^. Wu *et al*.^17^ suggested that XPC inactivation by promoter hypermethylation could increase the occurrence of p53 mutations in lung cancer patients^17^. Mechanistic studies have shown that XPC variants may act by influencing environmental or occupational exposures as the bulky DNA adducts formed by aromatic amines are repaired by NER. It appears that XPC variants increase UBC risk in complex with a number of environmental factors, including the habits of smoking or tobacco chewing.

Meta-analyses till date have analyzed XPC polymorphisms for correlation with overall cancer risk and the risk of specific cancers. Francisco *et al*.^23^ analyzed 33 studies and found that both the polymorphisms were significant risk factors for cancer and Ala499Val correlated particularly with an increased risk of UBC. In another meta-analysis on Ala499Val (5227 cases and 5959 controls) and Lys939Gln (9091 cases and 11553 controls), Zhang *et al*.^24^ reported that both the polymorphisms were unrelated to cancer risk. However, in stratified analysis by ethnicity, Ala499Val showed a correlation with UBC in Caucasians. A recent meta-analysis (He *et al*.^7^) analyzed 25708 cases and 30432 controls from 62 studies and reported that both the polymorphisms increased the risk of cancer significantly^7^. Stratification on the basis of cancer type showed a significant correlation of both the polymorphisms with UBC, particularly in the Asian populations^7^. Therefore, XPC polymorphisms appear to significantly increase cancer risk.

Among meta-analyses conducted specifically on UBC, Stern *et al*.^2^ reported only a weak association of A499V polymorphism with UBC. Later, Dai *et al*.^25^ performed a meta-analysis on 10 studies on Lys939Gln (3934 cases and 4269 controls) and five studies on Ala499Val (2113 cases and 2249 controls) and reported that both the polymorphisms increased the risk of UBC. Stratification analysis suggested a significant correlation of Lys939Gln in Asians and Ala499Val in Caucasians. In a similar analysis, Duo *et al*.^26^ performed a meta-analysis on 12 studies (4828 cases and 4890 controls) on Lys939Gln and reported a significant association of the substitution with UBC. Another recent meta-analysis (Wang *et al*.)^27^ on Ala499Val with a total of 7,674 subjects (seven studies) also suggested a significant association of this polymorphism with UBC risk, particularly in Caucasians. We conducted meta-analysis with the maximum numbers of studies followed by sensitivity analyses and trial sequential analyses, which suggested that Ala499Val is a risk factor and Lys939Gln is a neutral polymorphism with regard to UBC risk.

In conclusion, we observed a significant correlation of Ala499Val with UBC risk in Indian population, but Lys9393Gln was neutral. Meta-analysis and TSA corroborated these results, suggesting that Ala499Val was a risk factor for UBC and Lys939Gln was a neutral polymorphism. There is now sufficient evidence to conclude that XPC polymorphisms increase the risk of cancer, particularly UBC. From the literature review and a number of meta-analyses presented above, it can be postulated that Ala499Val is a more prominent risk factor for UBC and other cancers across a large number of populations; however, Lys939Gln may be largely neutral. Further analysis on the various ethnicities would validate these findings. We must admit some limitations of this meta-analysis. First, in the sub-group analysis, not all the ethnic populations were equally represented. Some of the sub-groups had higher numbers of studies in comparison to the others, which may bias the conclusion regarding the correlation status in each ethnic group. In stratified analysis, we could compare the risk factor (Ala499Val) only in Europeans and Americans due to a lack of studies on Indians and Chinese. Second, we found Ala499Val to be a significant risk factor only in the presence of smoking or tobacco chewing; therefore, it would have been ideal to adjust pooled OR value with respect to smoking and tobacco chewing. However, due to the lack of original data for each study, we could not adjust the pooled OR with respect to age, sex and environment.

## Material and Methods

### Sample collection

A total of 234 patients with histopathologically confirmed transitional cell carcinoma of bladder and 258 healthy control subjects were recruited from the Department of Urology at the King George’s Medical University (KGMU), Lucknow. Written informed consent was obtained from each participant for personal interviews and collection of blood samples for research purpose. Age, gender, and smoking status were registered for all the patients and controls. The Institutional Ethics Committee of the KGMU, Lucknow, approved the study (ref no. XLIX ECM A-/P14). The experiments were carried in accordance with the guidelines approved for research on human samples.

### DNA preparation and genotyping

Genomic DNA was extracted from the peripheral blood samples using a phenol-chloroform precipitation based method. The *XPC* polymorphisms were analyzed using polymerase chain reaction-restriction fragment length polymorphism (PCR-RFLP) and direct DNA sequencing methods. PCR was carried out in a reaction volume of 10 μl each in thin walled tubes, consisting of 1.0 μl of PCR buffer (10x)(New England Biolabs), 1.0 μl of 10 mM dNTPs (Genei), 2.0 pM of each of the forward and reverse primers, 1.0 unit of Taq DNA polymerase enzyme (New England Biolabs), and 40 ng of genomic DNA. PCR cycling was carried out in ABI Veriti thermal cycler (Applied Biosystems, USA). The primers used for K939Q polymorphism were; forward (5′ACCTGTCCAGAGTGAGGCAG3′) and reverse (5′TCAAAGGGTGAGTGGGCTTT3′) primers, and for Ala499Val were; forward-(5′TGGCCTCCAGGGTGTCTTAT3′) and reverse (5′ACTGTCAATGCCCACCACAT3′). PCR amplification conditions included denaturation at 95 °C for 5 minutes, followed by 35 cycles of denaturation at 95 °C for 30 seconds, annealing at 67 °C for 30 seconds, polymerization at 72 °C for 40 seconds, and a final stage of polymerization at 72 °C for 7 minutes.

PCR generated amplicons of 493bp and 390 bp for Lys939Gln and Ala499Val loci, respectively. PCR products were digested without further purification with one unit of *Pvu*II and *Aci*I restriction enzymes (New England Biolabs) for Lys939Gln and Ala499Val, respectively. After digestion, the samples were run on a 3% agarose gel and samples were classified as homozygous for Lys type (493 bp fragment), homozygous for the Gln type alleles (223 and 270 bp fragments), and heterozygous for Lys/Gln (493, 223, and 270 bp fragments). Similarly, in the case of Ala499Val, samples were classified as homozygous for Ala type (390 bp fragments), homozygous for Val type (222, 168) and heterozygous for Ala/Val alleles (390, 222 and 168 bp fragments) on the basis of banding pattern. Randomly selected 20% PCR products were sequenced by Sanger’s sequencing to confirm accuracy of the genotyping method.

### Statistical analysis

The distribution of genotypes was compared between cases and control using the Chi square test available at Vassarstats Online Calculator (http://faculty.vassar.edu/lowry/VassarStats.html). Dominant, co-dominant, recessive and allelic genetic models were adopted for statistical analysis. Gene-environment interactions were assessed by the stratification of subjects on the basis of smoking and tobacco chewing status. P value < 0.05 was considered to be statistically significant.

### Meta-Analysis

#### Search strategy

We conducted a search in the PubMed (http:// www.ncbi.nlm.nih.gov/), EMBASE, and Google Scholar databases with the keywords, XPC and bladder cancer, XPC and carcinoma bladder, DNA repair gene polymorphisms, and carcinoma of bladder, XPC and UBC and bladder neoplasm in different combinations to identify the articles published up to November 2015. The search was limited only to the articles published in English. The articles thus retrieved were examined by reading the titles and abstracts, and full text articles of potentially relevant publications were further checked for their suitability for inclusion in this meta-analysis.

Heterogeneity between the studies was assessed by the ‘Q’ statistic, which was considered statistically significant with P < 0.10. The heterogeneity was quantified by the I^2^ metric, which is independent of the number of studies used in meta-analysis (I^2^ < 25%, no heterogeneity; I^2^ = 25–50%, moderate heterogeneity; I^2^ > 50%, extreme heterogeneity). The combined odds ratio (OR) was estimated using the fixed effect (FE) model in the case of P_heterogeneity_ ≥ 0.10 and using the random-effects (RE) model in the case of P_heterogeneity_ < 0.10. High-resolution plots (forest plots) were generated to obtain the summary estimates. Publication bias was assessed from the distribution of studies on the funnel plot and statistically evaluated using Egger’s regression intercept test. Sensitivity analysis was conducted by removing the studies conducted on sample size less than 50 in either of the case control groups.

### Inclusion and exclusion criteria

Inclusion criteria comprised of the following: (i) Articles were original case–control or cohort studies on human subjects, (ii) The purpose of all the studies and statistical methods used were similar, and (iii) Articles had presented raw data necessary for the calculation of the crude odds ratio. Exclusion criteria comprised of the following: (i) Studies with obvious overlap of data with other included articles, (ii) Studies not providing enough information (raw data), and (iii) Studies not well described.

### Data extraction

Two authors analyzed the details of studies for meta-analysis. The following parameters from each study were recorded on a spreadsheet: the first author, the year of publication, the country of origin, ethnicity, and the number of cases and controls with genotype details.

### Pooled estimate

We have pooled data from our case-control study and other published studies on Ala499Val and Lys939Glnpolymorphisms. Meta-analysis was performed using the Comprehensive Meta-analysis software (CMA, version 2). Analysis was undertaken using dominant and recessive models. The strength of association was estimated by the OR and 95% CI.

### Trial sequential analysis (TSA)

The results of a meta-analysis may be biased by the presence of systematic errors (bias) or random errors (play of chance) due to sparse data and repeated significance testing. Trials with low methodological quality, publication bias and small sample size may generate a false P-value. Therefore, we used a novel statistical analysis software, TSA (Trial sequential analysis tool from Copenhagen Trial Unit, Center for Clinical Intervention Research, Denmark) that calculates the required information size by adjusting the significance level for sparse data and repeated testing to confirm statistical reliability of the data in a meta-analysis^28^. Some previous studies have reported that TSA outcomes are more reliable than those of the traditional meta-analyses^29^,^30^. In brief, the TSA tool calculates the required information size (number of samples) by considering an overall type –I error of 5% and type-II error of 20% and plots a two-sided graph, where red straight lines indicate the significance boundaries of the traditional meta-analysis, the blue line shows the cumulative Z-score, and red lines sloping inwards represent trial sequential monitoring boundaries with adjusted P-values.

## Additional Information

**How to cite this article**: Sankhwar, M. *et al*. Polymorphisms in the *XPC* gene affect urinary bladder cancer risk: a case-control study, meta-analyses and trial sequential analyses. *Sci. Rep.* **6**, 27018; doi: 10.1038/srep27018 (2016).

## Figures and Tables

**Table 1:** General characteristics of study subjects.

Characteristics	Cases 234 (%)	Controls 258 (%)
Age in years		
<30	8(3.41)	28(10.85)
30–40	32(13.67)	57(22.09)
41–50	52(22.22)	69(26.74)
51–60	63(26.92)	48(18.60)
>60	79(33.76)	56(21.70)
Mean ± SD	46.8 ± 27.61	51.60 ± 15.17
Sex		
Male	197(84.18)	201(77.90)
Female	37(15.81)	57(22.09)
Occupation		
Service	53(22.64)	66(25.58)
Business/Professional	48(20.51)	63(24.41)
Farmer	71(30.34)	47(18.21)
Labor	35(14.95)	19(7.36)
House wife	25(10.68)	45(17.44)
Student	2(0.008)	18(6.97)
Smoking		
Yes	127(54.27)	64(24.80)
No	107(45.72)	194(75.19)
Tobacco Chewing		
Yes	97(41.45)	69(26.74)
No	137(58.54)	189(73.25)

**Table 2:** Distribution of Ala499Val and Lys939Gln genotypes in patients and controls.

	Genotype/Allele	Ala499Val (C/T)		Lys939Gln (A/C)	
		Cases (%)	Controls (%)	Cases (%)	Controls (%)
Overall	11	52(22.22)	87(33.72)	63(26.92)	68(26.36)
	12	113(48.29)	112(43.41)	112(47.86)	114(44.18)
	22	69(29.49)	59(22.87)	59(5.21)	76(9.46)
Non-smokers	11	30(23.62)	16(25.00)	32(25.19)	15(23.44)
	12	78(61.42)	37(57.81)	62(48.81)	30(46.87)
	22	19(14.96)	11(17.19)	33(25.98)	19(29.69)
Smokers	11	22(20.56)	71(36.59)	31(28.97)	53(27.32)
	12	35(32.71)	75(38.66)	50(46.72)	84(43.30)
	22	50(46.73)	48(24.74)	26(24.30)	57(29.38)
Tobacco non-chewers	11	26(26.80)	19(27.53)	27(27.83)	18(26.08)
	12	48(49.48)	38(55.07)	42(43.30)	29(42.03)
	22	23(23.71)	12(17.39)	28(28.86)	22(31.88)
Tobacco chewers	11	26(18.98)	68(35.98)	36(26.28)	50(26.45)
	12	65(47.44)	74(39.15)	70(51.09)	85(44.97)
	22	46(33.57)	47(24.87)	31(22.63)	54(28.57)

**Table 3:** Statistical analysis of the genotype data between cases and controls.

	Genotype/Allele	*A499V* (C/T)	*K939Q*(A/C)
		OR (95%CI), p-value	OR (95%CI), p-value
Overall	11 vs 12	1.69(1.1–2.60), 0.017*	1.06(0.69–1.63), 0.79
	11 vs 22	1.96(1.20–3.19), 0.007*	0.84(0.52–1.36), 0.47
	12vs 22	1.16(0.75–1.79), 0.51	0.79(0.51–1.21), 0.28
	11 vs 12 + 22	1.78(1.19–2.66), 0.005*	0.97(0.65–1.45), 0.89
	22 vs11 + 12	1.41(0.94–2.11), 0.095	0.81(0.54–1.20), 0.29
Non-smokers	11 vs 12	1.12(0.55–2.31), 0.75	0.97(0.46–2.06), 0.92
	11 vs 22	0.92(0.35–2.40), 0.816	0.81(0.35–1.87), 0.63
	12vs 22	0.82(0.35–1.90), 0.64	0.84(0.41–1.71), 0.63
	11 vs 12 + 22	1.08(0.54–2.17), 0.84	0.91(0.45–1.84), 0.79
	22 vs11 + 12	0.85(0.38–1.91), 0.69	0.83(0.43–1.62), 0.59
Smokers	11 vs 12	1.51(0.81–2.81), 0.197	1.02(0.58–1.79), 1.00
	11 vs 22	3.36(1.81–6.26), < 0.0001*	0.78(0.41–1.48), 0.45
	12vs 22	2.23(1.27–3.92), 0.005*	0.77(0.43–1.37), 0.37
	11 vs 12 + 22	2.23(1.28–3.87), 0.004*	0.92(0.54–1.55), 0.76
	22 vs11 + 12	2.67(1.62–4.40), < 0.0001*	0.77(0.45–1.32), 0.35
Tobacco non-chewers	11 vs 12	0.92(0.45–1.91), 0.82	0.96(0.45–2.07), 0.92
	11 vs 22	1.40(0.56–3.50), 0.47	0.85(0.37–1.92), 0.69
	12vs 22	1.52(0.67–3.44), 0.32	0.88(0.42–1.82), 0.73
	11 vs 12 + 22	1.04(0.52–2.08), 0.92	0.91(0.45–1.84), 0.81
	22 vs11 + 12	1.48(0.68–3.22) 0.33	0.87(0.44–1.69), 0.68
Tobacco-chewers	11 vs 12	2.30(1.31–4.03), 0.003*	1.14(0.67–1.95), 0.62
	11 vs 22	2.56(1.39–4.70), 0.002*	0.80(0.43–1.47), 0.47
	12vs 22	1.11(0.66–1.88), 0.69	0.70(0.40–1.20), 0.19
	11 vs 12 + 22	2.40(1.43–4.04), 0.0008*	1.00(0.61–1.66), 1.00
	22 vs11 + 12	1.53(0.94–2.48), 0.086	0.73(0.44–1.22), 0.23

**Table 4:** Analysis of haplotype data for Ala499Val and Lys939Gln polymorphisms.

Haplotype Associations				
Block 1	Frequency	Case-control, ratio	Chi square	P value
CC	0.279	0.231, 0.322	10.253	0.0014
AC	0.258	0.233, 0.281	2.881	0.0896
AT	0.238	0.278, 0.202	7.721	0.0055
CT	0.225	0.259, 0.195	5.668	0.0173

**Table 5:** Demographic details and genotype data of studies included in the meta-analysis.

Study	Geographic region	Cases					Controls				
**Ala499Val**											
		Total	CC	CT	TT	CT + TT	Total	CC	CT	TT	CT + TT
Broberg *et al*.^20^	Europe	61	35	20	6	26	155	92	55	8	63
Wu *et al*.^17^	North America	603	355	216	32	248	590	333	232	25	257
Sak *et al*.^10^	Europe	538	279	202	57	259	565	317	210	38	248
Zhu *et al*.^18^	North America	546	323	193	30	223	549	310	215	24	239
Verdier *et al*.^6^	Europe	311	138	138	35	173	330	196	124	10	134
Liu *et al*.^13^	China	600	242	294	64	358	609	272	285	52	337
Present study	India	234	52	113	69	182	258	87	112	59	171
**Lys939Gln**											
		**Total**	**AA**	**AC**	**CC**	**AC + CC**	**Total**	**AA**	**AC**	**CC**	**AC + CC**
Sak *et al*.^8^	Europe	532	204	241	87	328	561	192	285	84	369
Garcia-Closas *et al*.^19^	Europe	1137	374	575	188	763	1138	411	536	191	727
Wu *et al*.^17^	USA	606	94	293	219	512	596	101	284	211	495
Zhu *et al*.^18^	USA	550	199	271	80	351	554	199	262	93	355
Wen *et al*.^4^*	China	304	119	NA	NA	185	90	37	NA	NA	53
Verdier *et al*.^6^	Europe	305	113	141	51	192	328	133	161	34	195
Rouissi *et al*.^21^	Europe	193	74	76	43	119	193	79	92	22	114
Zhi *et al*.^14^	China	302	118	136	48	184	311	138	138	35	173
Mittal *et al*.^15^	India	212	100	87	25	112	250	118	116	16	132
Liu *et al*.^13^	China	600	92	272	236	508	609	75	281	253	534
Present study	India	234	63	112	59	171	258	68	114	76	190

*NA = Not available, Wen *et al*.^4^ was included only in dominant model of meta-analysis.

**Table 6:** Summary of meta-analysis results.

XPC (Ala499Val)				XPC (Lys939Gln)		
	**OR (95%CI), P**	**(I^2^) %**	**Egger’s P**	**OR (95%CI), P**	**(I^2^) %**	**Egger’s P**
11 vs 12 + 22						
Overall	1.20 (0.98–1.48), 0.083	72.22	0.31	1.04 (0.95–1.13), 0.409	0.00	0.687
Europe	1.37 (1.07–1.76), 0.011*	62.31	–	1.05 (0.90–1.23), 0.521	37.49	–
America	0.90 (0.76–1.06), 0.22	0.00	–	1.03(0.85–1.25), 0.726	0.00	–
India	–	–	–	0.99 (0.75–1.29), 0.927	0.00	–
China	–	–	–	1.01 (0.80–1.27), 0.962	52.18	–
(11 vs 12)						
Overall	1.12 (0.92–1.35), 0.26	62.64	0.35	1.01 (0.92–1.11), 0.793	7.01	0.220
Europe	1.23 (0.99–1.52), 0.059	46.12	–	0.99 (0.83–1.19), 0.921	52.35	–
America	0.87 (0.73–1.03), 0.106	0.00	–	1.06 (0.87–1.30), 0.557	0.00	–
India	–	–	–	0.96 (0.72–1.28), 0.775	0.00	–
China	–	–	–	0.96 (0.72–1.28), 0.760	56.96	–
(11 vs 22)						
Overall	1.71 (1.26–2.31), 0.001*	52.01	0.34	1.14 (0.94–1.38), 0.193	54.52	0.080
Europe	2.46 (1.41–4.29), 0.001*	66.86	–	1.32 (0.93–1.88), 0.127	59.85	–
America	1.20 (0.81–1.77), 0.36	0.00	–	0.99 (0.77–1.26), 0.913	6.31	–
India	–	–	–	1.16 (0.66–2.06), 0.608	70.79	–
China	–	–	–	1.06 (0.64–1.74), 0.835	82.49	–
(12 vs 22)						
Overall	1.41 (1.16–1.72), 0.001*	8.00	0.08	1.13(0.94–1.36), 0.186	59.32	0.014
Europe	1.90 (1.32–2.75), 0.001*	20.39	–	1.35 (0.95–1.93), 0.094	74.76	–
America	1.38 (0.93–2.06), 0.110	0.00	–	0.94 (0.77–1.15), 0.562	0.00	-
India	–	–	–	1.16 (0.66–2.02), 0.615	81.87	–
China	–	–	–	1.12 (0.69–1.81), 0.652	41.17	–
(11 + 12 vs 22)						
Overall	1.54 (1.21–1.97), 0.001*	35.03	0.199	1.13 (0.95–1.34), 0.171	59.79	0.014
Europe	2.21 (1.39–3.50), 0.001*	55.20	–	1.34 (0.96–1.87), 0.089	71.11	–
America	1.27 (0.86–1.86), 0.224	0.00	–	0.96 (0.80–1.17), 0.700	0.00	–
India	–	–	–	1.14 (0.67–1.93), 0.639	80.39	–
China	–	–	–	1.11 (0.70–1.76), 0.648	70.63	–
